# The effects of caffeinated and decaffeinated coffee on sex hormone-binding globulin and endogenous sex hormone levels: a randomized controlled trial

**DOI:** 10.1186/1475-2891-11-86

**Published:** 2012-10-19

**Authors:** Nicole M Wedick, Christos S Mantzoros, Eric L Ding, Aoife M Brennan, Bernard Rosner, Eric B Rimm, Frank B Hu, Rob M van Dam

**Affiliations:** 1 Department of Nutrition, Harvard School of Public Health, 665 Huntington Ave, Boston, MA, 02115, USA; 2 Section of Endocrinology, Boston VA Healthcare System, Harvard Medical School, Boston, MA, USA; 3 Division of Endocrinology, Diabetes & Metabolism, Beth Israel Deaconess Medical Center, Harvard Medical School, Boston, MA, USA; 4 Channing Laboratory, Department of Medicine, Brigham and Women’s Hospital, and Harvard Medical School, Boston, MA, USA; 5 Department of Biostatistics, Harvard School of Public Health, Boston, MA, USA; 6 Department of Epidemiology, Harvard School of Public Health, Boston, MA, USA; 7 Saw Swee Hock School of Public Health and Department of Medicine, Yong Loo Lin School of Medicine, National University of Singapore, , Singapore

**Keywords:** Coffee, Sex hormones, Randomized trial

## Abstract

**Background:**

Findings from observational studies suggest that sex hormone-binding globulin (SHBG) and endogenous sex hormones may be mediators of the putative relation between coffee consumption and lower risk of type 2 diabetes. The objective of this study was to evaluate the effects of caffeinated and decaffeinated coffee on SHBG and sex hormone levels.

**Findings:**

After a two-week run-in phase with caffeine abstention, we conducted an 8-week parallel-arm randomized controlled trial. Healthy adults (n = 42) were recruited from the Boston community who were regular coffee consumers, nonsmokers, and overweight. Participants were randomized to five 6-ounce cups of caffeinated or decaffeinated instant coffee or water (control group) per day consumed with each meal, mid-morning, and mid-afternoon. The main outcome measures were SHBG and sex hormones [i.e., testosterone, estradiol, dehydroepiandrosterone sulfate].

No significant differences were found between treatment groups for any of the studied outcomes at week 8. At 4 weeks, decaffeinated coffee was associated with a borderline significant increase in SHBG in women, but not in men. At week 4, we also observed several differences in hormone concentrations between the treatment groups. Among men, consumption of caffeinated coffee increased total testosterone and decreased total and free estradiol. Among women, decaffeinated coffee decreased total and free testosterone and caffeinated coffee decreased total testosterone.

**Conclusions:**

Our data do not indicate a consistent effect of caffeinated coffee consumption on SHBG in men or women, however results should be interpreted with caution given the small sample size. This is the first randomized trial investigating the effects of caffeinated and decaffeinated coffee on SHBG and sex hormones and our findings necessitate further examination in a larger intervention trial.

## Introduction

Coffee consumption has been consistently associated with a lower risk of type 2 diabetes (T2DM), but the underlying mechanisms remain unclear. Data from observational studies suggest that sex hormone-binding globulin (SHBG) and endogenous sex hormones may modulate glycemia and risk of T2DM in men and women [1-5]. Caffeinated coffee consumption has been found to be associated with higher SHBG levels in data from cross-sectional studies in women [2,6-8]. It has been hypothesized that SHBG may be an intermediate pathway to explain the putative effect of coffee on lowering the risk of T2DM [3].

We conducted an 8-week parallel-arm randomized trial to determine the effects of caffeinated and decaffeinated coffee on risk factors for T2DM. To our knowledge, there have been no randomized trials to investigate this research question.

## Participants and methods

The details of this study have been previously described [9]. Briefly, eligible participants were overweight (body mass index 25–35 kg/m^2^), nonsmoking men and women aged 18 years or older who habitually consumed coffee (at least two cups per day). Exclusion criteria included the presence of diabetes, heart disease, stroke, hypertension, alcoholism or substance abuse, abnormal hepatic or renal function, gastro-esophageal reflux disease, a medical history of ulcers, or women planning a pregnancy or breastfeeding. Exclusions were made for individuals on medications for chronic health conditions.

Sixty-five adults were screened of which 11 were ineligible and 9 withdrew from the study prior to randomization. Three individuals did not continue the study after the baseline visit and were not included in the current analysis. The final study population included 14 men and 28 women. The study was approved by the institutional review boards of the Beth Israel Deaconess Medical Center and the Harvard School of Public Health and all participants provided written informed consent. The clinical trial registration number is NCT00305097.

After two weeks of caffeine abstention, participants attended the baseline visit in the morning after fasting overnight for at least 12 hours. Participants were randomized to either caffeinated coffee, decaffeinated coffee, or no coffee (control) treatment groups. Treatment assignments for the coffee arms were blinded to the study participants, investigators, and laboratory staff. Participants in the coffee treatment groups were given five two-gram portions of instant coffee per day (caffeinated or decaffeinated Nestlé’s Taster’s Choice®) to be mixed with approximately 6 ounces of boiling water and consumed with every meal and mid-morning and mid-afternoon. Non-caloric sweetener and non-dairy creamer were also provided. Participants in the control group were instructed to drink the equivalent amount of water at the same intervals throughout the day.

At baseline, week 4, and week 8, the study visits included a physical examination, anthropometric measurements, and a fasting blood draw. Sex hormone-binding globulin and all other endogenous sex hormones were measured via Access chemiluminescent immunoassay (Beckman Coulter, Fullerton, CA). Free testosterone and free estradiol were calculated using the Sodergard formula which is based on the law of mass action and assumptions of equilibrium binding [10].

All analyses were performed separately for men and women. Using general linear models, we evaluated the change from baseline in SHBG, testosterone (total and free), estradiol (total and free), testosterone to estradiol ratio, and DHEAs regressed on treatment group as a main effect with baseline log values of the dependent variable and age as additional covariates. Both covariates were grand mean centered to improve the interpretability of the estimates. Because SHBG and sex hormones did not follow a normal distribution, the variables were log-transformed and subsequently back-transformed to yield geometric means. Differences between caffeinated and decaffeinated coffee compared with the control group were based on linear contrasts. The adjusted geometric means with standard errors were reported by treatment, and 95% confidence intervals (CI) were computed. In addition, we calculated the difference between the treatment groups versus control for change from baseline. This yielded a ratio (or percentage when subtracting the value one and multiplying by 100), given the principles of logged numbers.

Statistical significance was evaluated at an alpha level of 0.05. The Statistical Analysis System version 9.1.3 was used for all analyses (SAS Institute, Cary, NC).

## Results

In men, mean concentrations were 25.3 nmol/L for SHBG, 411.3 ng/dL for testosterone, 8.0 ng/dL for free testosterone, 24.9 pg/mL for estradiol, 0.5 pg/mL for free estradiol, and 194.9 ng/mL for DHEAs. In women, mean concentrations were 51.5 nmol/L for SHBG, 35.9 ng/dL for testosterone, 0.5 ng/dL for free testosterone, 63.2 pg/mL for estradiol, 1.0 pg/mL for free estradiol, and 133.7 ng/mL for DHEAs. The baseline characteristics of the study population are shown in Table 1 according to treatment group. Ten women were postmenopausal. The average age was 40 years for both men (range 23–72 years) and women (18–69 years). Mean body mass index was 30.3 kg/m^2^ for men and 29.0 kg/m^2^ for women. All participants included in the analysis population completed the 8-week trial with the exception of one female participant who discontinued after the 4-week visit. Four non-serious adverse events were reported during the course of the intervention.

**Table 1:** Baseline characteristics for men and women by treatment group

	**MEN**			**WOMEN**		
	**Caffeinated**	**Decaffeinated**	**No Coffee**	**Caffeinated**	**Decaffeinated**	**No Coffee**
	**(N = 4)**	**(N = 4)**	**(N = 6 )**	**(N = 10)**	**(N = 9)**	**(N = 9 )**
Age (years)	37.2 (3.5)	35.0 (6.4)	47.3 (18.4)	40.0 (8.8)	43.2 (16.6)	38.4 (18.0)
Body mass index (kg/m^2^)	30.8 (1.9)	29.5 (2.7)	30.4 (1.3)	28.4 (1.9)	29.5 (2.2)	29.2 (2.5)
Waist circumference (cm)	97.7 (7.3)	102.9 (8.3)	102.0 (6.8)	85.9 (10.6)	90.9 (7.8)	88.3 (7.4)
Postmenopausal (n (%))	n/a	n/a	n/a	2 (20)	5 (56)	3 (33)
SHBG (nmol/L)	21.4 (13.7, 33.5)	19.5 (11.7, 32.5)	29.3 (18.2, 47.3)	52.6 (35.2, 78.6)	42.9 (28.6, 64.2)	38.1 (25.4, 57.0)
Testosterone (ng/dL)	387.9 (303.6, 495.7)	342.3 (259.0, 452.2)	475.5 (356.2, 634.6)	28.2 (19.2, 41.5)	27.3 (18.1, 41.1)	34.9 (23.2, 52.4)
Free testosterone (ng/dL)	8.0 (6.2, 10.3)	7.1 (5.3, 9.5)	8.0 (5.9, 10.7)	0.3 (0.2, 0.5)	0.3 (0.2, 0.5)	0.4 (0.2, 0.8)
Estradiol (pg/mL)	24.0 (17.0, 33.8)	21.7 (14.7, 32.1)	26.2 (17.5, 39.3)	47.4 (26.2, 85.8)	34.8 (18.6, 65.2)	47.7 (25.5, 89.4)
Free estradiol (pg/mL)	0.5 (0.3, 0.7)	0.4 (0.3, 0.7)	0.5 (0.3, 0.7)	0.8 (0.4, 1.4)	0.5 (0.3, 1.0)	0.8 (0.4, 1.5)
Testosterone : Estradiol	16.2 (11.4, 23.0)	15.8 (10.6, 23.5)	18.1 (12.0, 27.4)	0.6 (0.3, 1.2)	0.8 (0.4, 1.6)	0.7 (0.4, 1.5)
DHEAs (ng/mL)	184.1 (113.7, 298.1)	160.0 (92.4, 277.2)	161.7 (96.8, 270.1)	115.2 (82.7, 160.5)	98.1 (69.0, 139.3)	117.4 (82.7, 166.7)

n/a, not applicable; SHBG, sex hormone-binding globulin; DHEAs, dehydroepiandrosterone sulfate.

Data are arithmetic means (standard deviation) for continuous variables and percentages for categorical variables. Age-adjusted geometric means (95% confidence in intervals) are presented for hormone concentrations which did not follow a normal distribution.

Adjusted geometric means followed by percent change from baseline estimates for the endpoints are shown for men (Table 2) and women (Table 3). At the final study visit (week 8) there were no significant differences for any of the outcomes. In addition, we did not observe an effect of coffee intake on SHBG levels in men, although a borderline significant increase for decaffeinated coffee was observed among women [difference in change from baseline (CFB): 38%; 95% CI: 1%, 88%; p = 0.04] compared with consuming no coffee at week 4. In contrast, several significant differences between the treatment groups were found at week 4. Among men, consumption of caffeinated coffee increased total testosterone (CFB: 67%; 95% CI: 4%, 168%; p = 0.04) and decreased total and free estradiol (CFB total: -47%; 95% CI: -19%, -65%; p = 0.01 and CFB free: -43%; 95% CI: -10%, -64%; p = 0.02). Among women, decaffeinated coffee decreased total and free testosterone (CFB total: -60%; 95% CI: -24%, -79%; p = 0.01 and CFB free: -68%: 95% CI: -26%, -86%; p = 0.01) and caffeinated coffee decreased total testosterone (p = 0.04). The ratio of testosterone to estradiol, a potential marker for aromatase activity, was significantly increased in men in the caffeinated coffee group at week 4 (CFB: 189%; 95% CI: 39%, 502%; p = 0.01) whereas no significant differences were observed for women. We did not observe any significant effects for DHEAs in either men or women.

**Table 2:** Sex hormone-binding globulin and endogenous sex hormones by treatment group at week 4 and week 8 in men

	**Week 4**			**Week 8**		
	**Value (95% confidence interval)**			**Value (95% confidence interval)**		
	**Caffeinated**	**Decaffeinated**	**No Coffee**	**Caffeinated**	**Decaffeinated**	**No Coffee**
	**Coffee**	**Coffee**		**Coffee**	**Coffee**	**Coffee**
	**(N = 4)**	**(N = 4)**	**(N = 6)**	**(N = 4)**	**(N = 4)**	**(N = 6)**
SHBG (nmol/L)						
Geometric means^†^	20.7 (15.2, 28.1)	23.9 (16.7, 34.2)	23.0 (16.3, 32.4)	23.1 (19.4, 27.5)	25.0 (20.4, 30.6)	23.1 (19.0, 28.0)
Difference in CFB (%)^‡^	−10.2 (−44.7, 45.7)	3.8 (−39.3, 77.4)	Reference	0.2 (−23.8, 31.8)	8.2 (−20.1, 46.5)	Reference
Testosterone (ng/dL)						
Geometric means^†^	546.2 (412.8, 722.7)	493.6 (350.5, 695.1)	327.4 (227.7, 470.8)	403.8 (349.8, 466.0)	386.1 (324.0, 460.1)	373.1 (309.8, 449.5)
Difference in CFB (%)^‡^	66.8 (3.9, 167.9) ^a^	50.8 (−13.1, 161.6)	Reference	8.2 (−15.1, 37.9)	3.5 (−22.0, 37.2)	Reference
Free testosterone (ng/dL)						
Geometric means^†^	11.7 (7.0, 19.6)	9.2 (5.1, 16.8)	6.6 (3.6, 12.2)	8.0 (6.6, 9.6)	7.6 (6.1, 9.5)	7.0 (5.6, 8.7)
Difference in CFB (%)^‡^	76.0 (−21.8, 295.9)	39.0 (−42.8, 238.0)	Reference	14.5 (−14.9, 54.1)	9.7 (−20.8, 51.9)	Reference
Estradiol (pg/mL)						
Geometric means^†^	22.5 (17.2, 29.5)	35.9 (26.3, 49.0)	42.6 (30.9, 58.7)	25.2 (19.3, 32.8)	27.1 (20.0, 36.9)	20.7 (15.1, 28.4)
Difference in CFB (%)^‡^	−47.1 (−65.5, -19.1) ^b^	−15.8 (−47.3, 34.5)	Reference	21.7 (−20.0, 85.3)	31.2 (−17.4, 108.2)	Reference
Free estradiol (pg/mL)						
Geometric means^†^	0.4 (0.3, 0.6)	0.7 (0.5, 0.9)	0.8 (0.6, 1.1)	0.5 (0.4, 0.6)	0.5 (0.4, 0.71)	0.4 (0.3, 0.6)
Difference in CFB (%)^‡^	−42.8 (−63.7, -9.9) ^a^	−12.7 (−46.5, 42.4)	Reference	21.4 (−21.9, 88.8)	31.1 (−18.5, 110.9)	Reference
Testosterone : Estradiol						
Geometric means^†^	24.2 (15.2, 38.4)	12.6 (7.5, 21.4)	8.4 (4.8, 14.5)	16.1 (12.2, 21.3)	14.6 (10.6, 20.1)	17.5 (12.5, 24.4)
Difference in CFB (%)^‡^	188.8 (38.5, 502.1) ^b^	50.9 (−31.7, 233.6)	Reference	−8.0 (−41.1, 43.8)	−16.6 (−48.5, 35.1)	Reference
DHEAs (ng/mL)						
Geometric means^†^	178.0 (161.1, 196.8)	165.4 (147.7, 185.3)	159.8 (143.7, 177.6)	193.9 (168.1, 223.7)	164.1 (139.6, 193.0)	162.9 (140.1, 189.5)
Difference in CFB (%)^‡^	11.4 (−4.2, 29.5)	3.5 (−12.0, 21.7)	Reference	19.0 (−4.0, 47.6)	0.7 (−20.1, 27.0)	Reference

*SHBG*, sex hormone-binding globulin; *CFB*, change from baseline; *DHEAs*, dehydroepiandrosterone sulfate.

^†^ Geometric mean as the dependent variable with treatment as a main effect, and baseline log value (grand mean centered) and age (grand mean centered) as covariates.

^‡^ Difference in change from baseline compared to No Coffee (i.e., ratio of ratios) adjusted for the above covariates.

^a^ p < 0.05 compared to No Coffee.

^b^ p < 0.01 compared to No Coffee.

**Table 3:** Sex hormone-binding globulin and endogenous sex hormones by treatment group at week 4 and week 8 in women

	**Week 4**			**Week 8**		
	**Value (95% confidence interval)**			**Value (95% confidence interval)**		
	**Caffeinated**	**Decaffeinated**	**No Coffee**	**Caffeinated**	**Decaffeinated**	**No Coffee**
	**Coffee**	**Coffee**	**Coffee**	**Coffee**	**Coffee**	
	**(N = 10)**	**(N = 9)**	**(N = 9)**	**(N = 10)**	**(N = 9)**	**(N = 9)**
SHBG (nmol/L)						
Geometric means^†^	43.8 (35.2, 54.5)	52.8 (42.5, 65.5)	38.2 (30.7, 47.6)	44.2 (37.3, 52.4)	45.8 (38.7, 54.1)	44.2 (37.3, 52.4)
Difference in CFB (%)^‡^	14.6 (−16.2, 56.7)	38.0 (1.5, 87.7) ^a^	Reference	−0.0 (−21.6, 27.4)	3.6 (−18.4, 31.5)	Reference
Testosterone (ng/dL)						
Geometric means^†^	51.3 (33.5, 78.6)	39.0 (24.8, 61.4)	98.3 (62.3, 155.2)	35.1 (26.2, 47.0)	32.4 (23.8, 44.2)	37.3 (27.3, 51.0)
Difference in CFB (%)^‡^	−47.8 (−72.1, -2.3) ^a^	−60.3 (−79.3, -24.0) ^b^	Reference	−5.9 (−38.7, 44.3)	−13.1 (−44.3, 35.5)	Reference
Free testosterone (ng/dL)						
Geometric means^†^	0.6 (0.3, 1.0)	0.4 (0.2, 0.7)	1.2 (0.7, 2.1)	0.4 (0.3, 0.6)	0.3 (0.2, 0.5)	0.4 (0.3, 0.6)
Difference in CFB (%)^‡^	−51.1 (−78.7, 12.0)	−67.8 (−86.0, -26.2) ^b^	Reference	−6.0 (−47.5, 68.5)	−13.3 (−51.6, 55.4)	Reference
Estradiol (pg/mL)						
Geometric means^†^	37.9 (26.5, 54.2)	42.6 (29.0, 62.3)	42.9 (28.7, 64.1)	41.8 (24.8, 70.6)	39.7 (22.7, 69.4)	40.6 (23.3, 70.6)
Difference in CFB (%)^‡^	−11.8 (−48.4, 50.8)	−0.9 (−43.2, 73.2)	Reference	3.2 (−51.7, 120.5)	−2.1 (−55.8, 116.6)	Reference
Free estradiol (pg/mL)						
Geometric means^†^	0.6 (0.4, 0.9)	0.6 (0.4, 0.9)	0.7 (0.5, 1.1)	0.7 (0.4, 1.1)	0.6 (0.3, 1.0)	0.6 (0.4, 1.1)
Difference in CFB (%)^‡^	−19.3 (−56.1, 48.3)	−13.4 (−53.5, 61.3)	Reference	2.9 (−52.2, 121.6)	−5.2 (−56.8, 108.2)	Reference
Testosterone : Estradiol						
Geometric means^†^	1.4 (0.8, 2.3)	1.0 (0.6, 1.7)	2.1 (1.2, 3.8)	0.8 (0.5, 1.5)	0.8 (0.4, 1.5)	0.9 (0.5, 1.7)
Difference in CFB (%)^‡^	−36.0 (−71.0, 41.4)	−54.1 (−79.6, 3.3)	Reference	−9.9 (−61.7, 111.9)	−9.6 (−62.6, 118.3)	Reference
DHEAs (ng/mL)						
Geometric means^†^	113.8 (101.0, 128.1)	101.7 (89.6, 115.5)	100.0 (87.5, 114.4)	111.7 (101.0, 123.6)	102.9 (92.3, 114.6)	120.4 (108.2, 134.1)
Difference in CFB (%)^‡^	13.7 (−4.9, 36.0)	1.7 (−15.6, 22.5)	Reference	−7.2 (−19.9, 7.4)	−14.6 (−26.7, -0.4)	Reference

*SHBG*, sex hormone-binding globulin; *CFB*, change from baseline; *DHEAs*, dehydroepiandrosterone sulfate.

^†^ Geometric mean as the dependent variable with treatment as a main effect, and baseline log value (grand mean centered) and age (grand mean centered) as covariates.

^‡^ Difference in change from baseline compared to No Coffee (i.e., ratio of ratios) adjusted for the above covariates.

^a^ p < 0.05 compared to No Coffee.

^b^ p < 0.01 compared to No Coffee.

## Discussion

In this randomized controlled trial with a caffeinated and decaffeinated coffee intervention, we did not find evidence of a consistent effect on SHBG levels in overweight men or women. All significant effects for SHBG and the hormone measurements were limited to the week 4 visit with no significant effects observed at the time of the final week 8 visit.

Previous studies on coffee or caffeine consumption in relation to SHBG and sex hormone concentrations all had a cross-sectional design and have been almost exclusively conducted in women. The Additional file 1: Table S1 shows the characteristics and findings from these studies. Our results for caffeinated coffee and SHBG are consistent with two previous cross-sectional studies [11,12] which did not find an association with consumption of caffeine or caffeinated coffee, whereas other studies did detect direct associations [2,3,6-8]. In contrast to our findings for caffeinated coffee, we found slightly elevated SHBG levels in the decaffeinated group as compared with the control group at week 4 in women, but this observation was limited to women and not observed at week 8 and may well represent a chance finding. Few studies have specifically studied decaffeinated coffee, but the Women’s Health Study [3] observed no association between decaffeinated coffee and SHBG.

Our results in women of a decrease in total testosterone levels at week 4 in both caffeinated and decaffeinated arms are not consistent with the lack of association between coffee consumption and testosterone in previous observational studies [2,3,7]. We did not observe a significant effect of coffee consumption on estradiol concentrations among the women in our trial. This finding agrees with four cross-sectional studies that also found no association between caffeinated coffee consumption and estradiol [3,6,7,11]. In contrast, an inverse association between coffee consumption and luteal estradiol and luteal free estradiol was observed among premenopausal women [2] and a direct association was observed for follicular estradiol in another study among premenopausal women [12]. It is currently unclear whether the discrepancy between our findings and previous studies is due to the limited power or duration of our trial or methodological limitations of the cross-sectional studies.

As mentioned previously, little data has been published on coffee consumption and SHBG or sex hormones in men. Our finding that caffeinated coffee, but not decaffeinated coffee, significantly increased total testosterone and decreased both total and free estradiol after 4 weeks suggests that caffeine may act as an aromatase (or *CYP19*) inhibitor. One intervention trial [13] found that consumption of two cups of instant coffee had no acute effect on testosterone or estradiol concentrations after 30 minutes.

This is the first randomized controlled trial investigating the effects of caffeinated and decaffeinated coffee on SHBG and sex hormones. Attrition was low among participants and non-fasting blood samples measured for caffeine and its major metabolites at the 6-week visit indicated that compliance was high. Our study also had several limitations that need to be considered. Most notably, our study has a small sample size which may have limited our ability to detect modest effects on SHBG and sex hormone levels. Thus, findings should be interpreted with caution and require confirmation in larger trials. In addition, given the small sample size, stratifying analyses by menopausal status was not appropriate. Inclusion of age in the analysis of covariance models was an attempt to address this issue. From the evidence to date, it is clear that heterogeneity among the observational studies in timing of the hormone measurements in women has most likely led to some of the divergent findings. We inquired about the last menstrual period or menopausal status, in addition to having the two follow-up visits timed approximately four weeks apart to reduce the variation in measurement for sex hormones by follicular and luteal cycle timing. However, it is plausible that inadequate control for menopausal status attenuated our results given the variability in the women’s ages and that, for example, the majority of postmenopausal women were in the decaffeinated coffee group. In our trial, lack of finding any significant effects at the 8-week visit for any of the measurements lends to the hypothesis that habituation may have occurred by the time of the final 8-week visit. Longer randomized trials with larger sample size will be necessary to elucidate the temporality of the potential effects.

In this randomized controlled trial with caffeinated and decaffeinated coffee interventions, we did not find evidence of a consistent effect on SHBG levels in overweight men or women. This contrasts with the beneficial effects of coffee consumption on adiponectin and fetuin-A levels previously reported in this trial [9], suggesting that the SHBG level is not the major intermediate of the putative effect of coffee consumption on a lower risk of T2DM. Our findings necessitate further examination in a larger intervention trial of the effects of coffee on sex hormones to elucidate if this is a potential intermediary mechanism to explain the beneficial effects observed of coffee intake and T2DM.

## Competing interests

The authors declare that they have no competing interests.

## Authors’ contributions

NW contributed towards the acquisition, analysis and interpretation of the data, drafted the manuscript, and critically reviewed the manuscript. CM contributed towards the study concept and design, the acquisition, analysis and interpretation of the data, and critically reviewed the manuscript. ED contributed towards the analysis and interpretation of the data, and critically reviewed the manuscript. AB contributed towards the study concept and design, the acquisition and interpretation of the data, and critically reviewed the manuscript. BR contributed towards the analysis and interpretation of the data, and critically reviewed the manuscript. ER contributed towards the analysis and interpretation of the data, and critically reviewed the manuscript. FH contributed towards the study concept and design, the analysis and interpretation of the data, and critically reviewed the manuscript. RVD contributed towards the study concept and design, the acquisition, analysis and interpretation of the data, drafted the manuscript, critically reviewed the manuscript, and obtained funding for the study. All authors have read and approved the final manuscript.

## Supplementary Material

Additional file 1 **Table S1.** Summary of the observational evidence for the relation between coffee and caffeine intake, SHBG and sex hormone levels. (DOC 47 kb) Click here for file
