# Supplementary material for: The effects of caffeinated and decaffeinated coffee on sex hormone-binding globulin and endogenous sex hormone levels: a randomized controlled trial
Source: Nutr J. 2012 Oct 19;11:86. doi: 10.1186/1475-2891-11-86 (PMC3502342; doi:10.1186/1475-2891-11-86)
Supplement: Additional file 1 — Table S1. Summary of the observational evidence for the relation between coffee and caffeine intake, SHBG and sex hormone levels. (DOC 47 kb) [file 1475-2891-11-86-S1.doc]

| **Online Supplemental Table. Summary of the observational evidence for the relation between coffee and caffeine intake, SHBG and sex hormone levels.** | | | | | |
| --- | --- | --- | --- | --- | --- |
|  | **Reference, Year** | **Study Population / Design** | **Exposure** | **Outcome** | **Risk estimates** |
| 1 | London et al., 1991 | MA Women’s Health Study  n=352  perimenopausal women (50-60 yr)  / cross-sectional | FFQ  Caffeine  (mean=315 mg/day) | 1) SHBG  2) Estradiol  3) % free estradiol  4) Estrone | 1) r_s_ = 0.13, p <0.05  2) NS (p-value not given)  3) r_s_ = -0.15, p <0.01  4) NS (p-value not given)  adj. for age, calories, bmi, smoking |
| 2 | Cooper et al., 1992 | Rochester Epidemiology Project  n=290  women (30+ yr)  / cross-sectional | 7-day diet record  Caffeine (coffee, tea, other caff bev)  (median=210 mg/day; ~82% from coffee) | 1) SHBG  2) Estradiol  3) Estrone | All correlations non-significant once age-adjusted. |
| 3 | Ferrini et al., 1996 | Rancho Bernardo Study  n=728  postmenopausal women (42-90 yr)  / cross-sectional | FFQ  Caffeine (coffee, tea, soda)  (range: 0-881 mg/day) | 1) SHBG  2) Estradiol  3) bio Estradiol  4) Estrone  5) Testosterone  6) bio Testosterone | 1) r = 0.09; p = 0.03  2) NS  3) NS  4) r = 0.26; p = 0.05 (at high caffeine doses)  5) NS  6) r = -0.10, p = 0.02  adj. for age, bmi, WHR, smoking, alcohol, exercise |
| 4 | Nagata et al., 1998 | Gifu, Japan  n=50  premenopausal women (21-42 yr)  / cross-sectional | Diet questionnaire validated against 12 diet records administered over one-month intervals.  (mean=182 mg/day) | 1) SHBG  2) Estradiol  3) Estradiol:SHBG  measured at Days 11 and 22 of menstrual cycle | Day 11  1) r_s_ = 0.30, p <0.05 (caffeinated coffee)  2) r_s_ = -0.35, p <0.05  (green tea)  3) r_s_ = -0.32, p = 0.04 (green tea) and  r_s_ = -0.35, p = 0.02 (total caffeine)  Day 22  1) r_s_ = 0.32, p <0.05 (total caffeine)  adj. for age, BMI, and cycle length  All other correlations NS. |
| 5 | Lucero et al., 2001 | 7 communities in Greater Boston area  n=498  premenopausal women (26-45 yr)  / cross-sectional | FFQ  Caffeine  Coffee intake | Follicular phase  1) SHBG  2) Estradiol | 1) NS  2) 28.2 pg/mL (≤100 mg/d) versus  45.2 pg/mL (≥500 mg/d)  β = 0.0005; p = 0.010  for caffeine (mg/day)  adj. for age, BMI, calories, smoking, alcohol, cholesterol intake, day of sampling |
| 6 | Kotsopoulos et al., 2009 | Nurses Health Study I  n=713  postmenopausal women  Nurses Health Study II  n=524  premenopausal women  / cross-sectional | FFQ  Caffeine | 1) SHBG  2) Estradiol  3) free Estradiol  4) Estrone  5) Testosterone  6) free Testosterone  7) DHEAs | p for trend < 0.05 for:  premenopausal women  inverse associations  2) luteal estradiol  3) luteal free estradiol  postmenopausal women  1) borderline positive association among caffeine (not coffee) quartile analysis  adj. for age at blood draw and first birth, bmi, assay batch, fasting status, time of blood draw, smoking, alcohol, exercise |
| 7 | Goto et al., 2010 | Women’s Health Study  n=718  postmenopausal women  / case-control (1:1) | SFFQ  Caffeine | 1) SHBG  2) Estradiol  3) Testosterone  4) DHEAs | 1) positive associations (p for trend = 0.01) for caffeinated coffee and total caffeine (p for trend = 0.02)  2) NS  3) NS  4) NS  Also, NS for decaffeinated coffee or tea intake.  Matched on age, race, duration of follow-up, time of blood draw in addition to smoking, alcohol, exercise, bmi, and calories. |

NS indicates non-significant findings; SHBG = sex hormone-binding globulin; bio = bioavailable; DHEAs, dehydroepiandrosterone sulfate.
